# Potential of green synthesized titanium dioxide nanoparticles for enhancing seedling emergence, vigor and tolerance indices and DPPH free radical scavenging in two varieties of soybean under salinity stress

**DOI:** 10.1186/s12870-022-03945-7

**Published:** 2022-12-02

**Authors:** Hanan Abdalla, Marwa H. Adarosy, Hegazy S. Hegazy, Reda E. Abdelhameed

**Affiliations:** Botany and Microbiology Department, Faculty of Science, Zagazig University, Zagazig, 44519 Egypt

**Keywords:** Green synthesis, TiO_2_ nanoparticles, DPPH free radical, Salinity, Soybean, Vigor index

## Abstract

**Background:**

Considering titanium dioxide nanoparticles (TiO_2_ NPs) role in plant growth and especially in plant tolerance against abiotic stress, in the present work, TiO_2_ NPs were green synthesized using an aqueous solution of *Aloe vera* leaf extract as a capping agent and titanium tetrachloride as a precursor. These green synthesized TiO_2_ NPs were characterized using different techniques: UV spectrophotometer, scanning electron microscopy (SEM), transmission electron microscopy (TEM), Fourier transform infrared (FTIR) spectroscopy and X-ray diffraction (XRD). Results revealed that synthesized TiO_2_ NPs possess a tetragonal morphology with a size ranging from 10 to 25 nm. Additionally, the present work evaluated the effects of three concentrations of TiO_2_ NPs (0, 30 and 50 ppm) and six NaCl concentrations (0, 25, 50, 100, 150 and 200 mM) and their interactions with respect to germination parameters, vigor indices, oxidative stress and DPPH free radical scavenging of two varieties of soybean (*Glycine max* L. var. 22 and 35).

**Results:**

Results demonstrated that all germination traits and vigor indices were negatively affected under all salinity levels. Also, the contents of hydrogen peroxide (H2O2) and malondialdehyde (MDA) were significantly increased by increasing the NaCl concentrations in two soybean varieties. Most interestingly, TiO2 NPs (30 ppm) mediated positive effects on germination parameters, reducing H2O2 and MDA contents by enhancing antioxidant (decreasing IC50) whereas 50 ppm showed an intermediate response under both control and saline soil conditions.

**Conclusion:**

Our findings demonstrate the growth enhancement effects of TiO_2_ NPs application as well as its ameliorative potential in dealing with salinity.

## Highlights

• Pure synthesis of TiO_2_ NPs using the leaf extract of *A. vera* as a reducing and stabilizing agent.

• The synthesized TiO_2_ NPs have a tetragonal shape with a size range from 10 to 25 nm.

• Salinity reduced germination attributes and vigor indices of two soybean varieties.

• H_2_O_2_ and MDA contents increased by increasing NaCl concentrations

• TiO_2_ NPs induced salt tolerance in soybean by enhancing germination traits, DPPH free radical scavenging (Less IC50) and reducing H_2_O_2_ and MDA contents

## Background

Salinity, as one of major abiotic stresses, not only restricts plant growth but also retards developmental processes in important crops [1–4]. Also, salinity obstructs germination by creating a harmful effect on the germination of seeds of many crops by forming an osmotic pressure on the outer side of the seed which inhibits water absorption [5]. At a global level, about 1,125 million hectares of agricultural lands were harshly impacted by salt stress, which resulted in reduced agricultural production and crop yield [6]. Commonly, salinity affects metabolic processes, such as protein synthesis, carbohydrates and lipid metabolism through osmotic and ionic stress [7]. Moreover, inhibition of cellular and membrane dysfunction occurs under salt stress owing to a greater accumulation of sodium ion (Na^+^) in plant tissues which as well initiates osmotic stress leading to a water deficit in cells and consequently lessens water potential [8].

Nowadays, environmental safety is an emerging global challenge in the twenty-first century for environmental protection and sustainable development. So, an increase in salt tolerance of crops is essential to enhance food production in several regions of the world [3]. In this respect, to mitigate the harmful effects of saline stress, various methods have been adopted to adjust the ion equilibrium and osmotic homeostasis to overcome the impact of salt injury [9]. Among different measures, an efficient and simple approach to recover plant performance under stressful conditions is seed priming with nanoparticles (NPs) [10–12]. Seed priming is generally defined as the controlled hydration of the seeds to the level that allows pre-germinative metabolic activity to continue while averting the surfacing of the radicle. NPs are known as stimulating agents for plant growth modulating the physiological, biochemical and physicochemical pathways [13]. One of these, TiO_2_ is a well-known NP has a wide range of applications in cleaning air products, skincare products, and cosmetics, and for organic matter decomposition in wastewater [14]. Most interestingly that TiO_2_ NPs have several insightful impacts on the crop features and play a vital role not only in plant growth but also in development due to their chemical stability and non-toxic nature [15]. Mahmoodzadeh et al. [16] reported elevated levels of germination and improved radicle and plumule development in canola seedlings when treated with TiO_2_ NPs. As well, TiO_2_ NPs increased growth and production attributes like yield in wheat seedlings thriving under water stress [17]. Wu et al. [18] reported a positive effect of TiO_2_ NPs on seed germination, when bombarding lettuce seeds via the electrospray technique, even at low pH or using aged seeds.

Likewise, the beneficial impact of TiO_2_ NPs on seed germination of chickpea (*Cicer arietinum*) and in fodder crops such as berseem and oat has been reported [19, 20]. By contrast, TiO_2_ NPs toxicity on plants has been reported as well, with delayed germination [21], this toxicity like various other NPs mainly depends upon their concentration, type, size, and duration of application, assuming that low concentrations are beneficial and high concentrations are toxic. For example, wheat plants subjected to different concentrations of TiO_2_-NPs indicated that the use of these NPs in a suitable concentration improved germination indices in comparison to control plants, whereas high concentrations had inhibitory effects on wheat germination [22].

Green NP synthesis has emerged as a new trend in nanotechnology development that is both safer and more eco-friendly. Biological agents as sustainable resources like extracts of the plant (flower, leaf, stem, seeds and bark), bacteria, fungi, algae, and actinomycetes are used in the green synthesis process instead of dangerous chemical agents, which might have negative consequences [23, 24]. One of the benefits of green synthesis is its cost-effectiveness, as the approach uses very inexpensive components. Another benefit is the method's simplicity due to the one-step process [25]. Indeed, the use of plant extracts can be more beneficial for NP synthesis than bacterial and chemical methods because of the lack of any threat of bacterial and dangerous chemical contamination with wider implications and easiness. Several plant extracts have been used in previous green synthesis studies due to their ease of production such as *Ficus religiosa* [26], *Citrus limon*, *C. reticulate*, *C. sinensis* [27], *Aloe vera* [28, 29] and *Moringa oleifera* [30, 31].

Soybean (*Glycine max* L.) is a globally important crop that provides protein and oil for a wide array of products. By weight, soybean seed is made up of roughly 40% protein, 20% oil, 35% carbohydrate and 5% ash [32] and most soybeans are processed for oil and protein meal. Studies of soybean have shown that high salinity may delay or inhibit germination, cause reductions in plant height, leaf size, biomass, number of branches, number of pods and weight of seeds [33, 34]. A major reduction in any one of these categories can severely limit the yield potential of the soybean crop and have catastrophic effects on the farmer’s financial return. Not only does salt stress negatively impact germination and growth of soybean plants, but this abiotic stress can also cause a reduction in the agronomic quality of beans harvested from salt-stressed soybean plants. In the present research, the prime aim is to investigate the effects of soybean seed priming with TiO_2_ NPs on overall seed development including both seed germination, seedling development and oxidative stress markers under different salt concentrations, then determine the optimized concentration of TiO_2_ NPs for seed priming.

## Results and discussion

### Phytochemical study and HPLC analysis

**Fig. 1:**
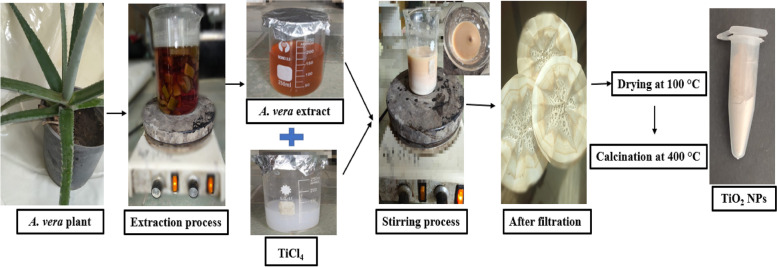
A schematic procedure for the preparation of aqueous leaf extract of *A. vera* and synthesis of TiO_2_ NPs using this extract

A schematic procedure for the preparation of *A. vera* aqueous leaf extract is presented in Fig. 1. As the biomaterials and phytochemicals included in the plant extract are completely necessary for the green creation of NPs to avoid the usage of chemical stabilizers, the phytochemical analysis of this extract was carried out, and the results are showed in Table 1. The *A. vera* aqueous leaf extract was shown to be an excellent source of secondary metabolites from the phytochemical screening, where phenolics, flavonoids, proteins, alkaloids, saponins, and tannins were detected. It was noticed that the aqueous extract of *A. vera* contained high polyphenols, which may be the cause of the observed biological activity in NPs synthesis. These phytoconstituents are involved as bioreductants and stabilizers during the process of green synthesis, as previously mentioned by Selim et al. [35]. Moreover, Ahmad et al**.** [36] showed that the terpenoids, flavones, ketones, aldehydes and amides, commonly found in the bioactive chemicals of plant extracts, are added as reducing and stabilizing agents during the NPs synthesis.

**Table 1:** Qualitative phytochemical screening of aqueous leaf extract of *A. vera*

Phytoconstituents	Aqueous *A. vera* leaf extract
Flavonoids	** + **
Phenolics	** + + **
Saponins	** + + **
Alkaloids	** + **
Tannins	** + **
Glycosides	**-**
Coumarins	** + **
Proteins	** + **

Highly present ‘ +  + ’, present ‘ + ’, absent ‘ − ’

**Fig. 2:**
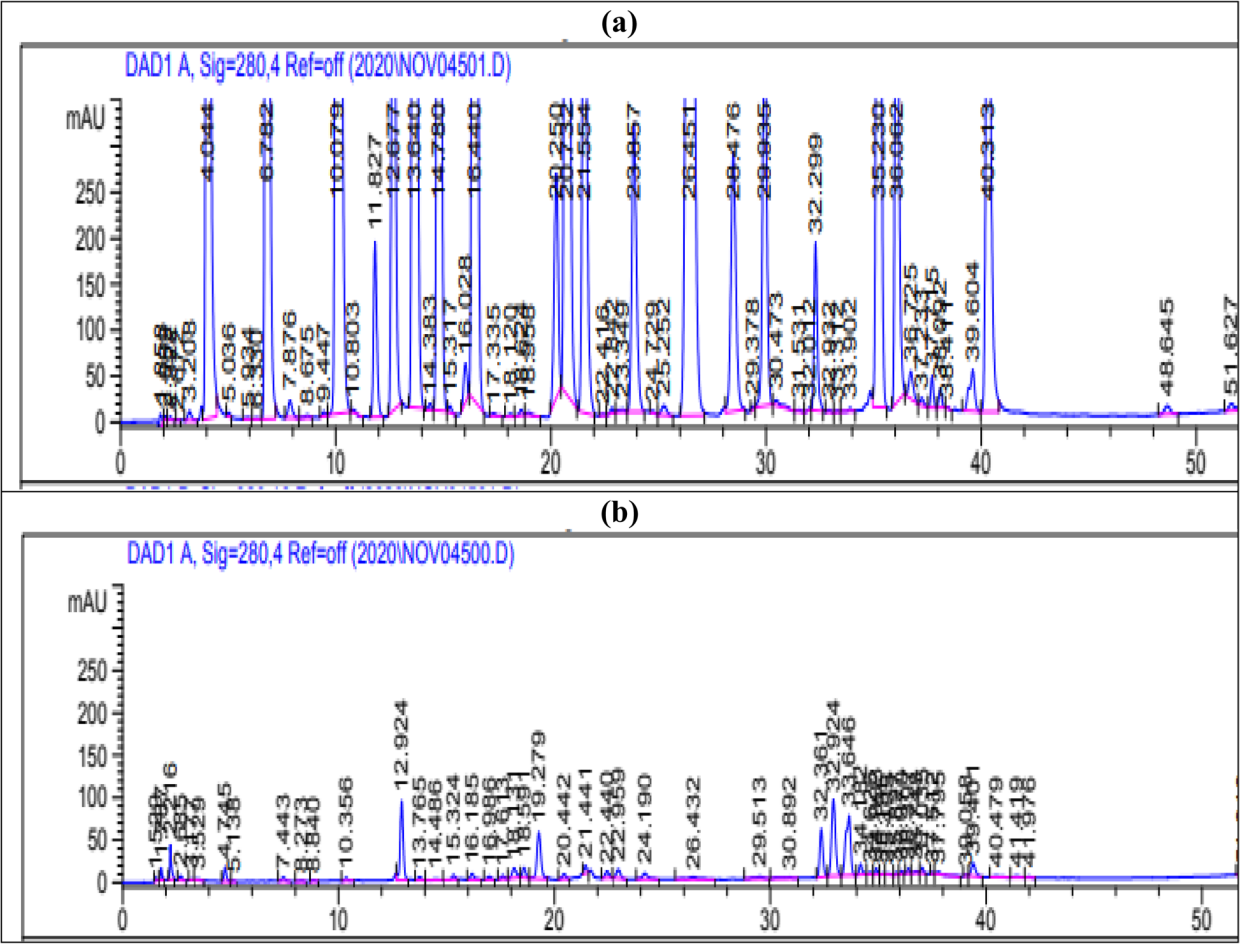
HPLC chromatogram: (**a**) standard mixture of polyphenolic compounds; (**b**) aqueous leaf extract of *A. vera*

HPLC analysis detected the presence of polyphenolics such as chlorogenic acid, rutin, *p*-coumaric acid, *p*-hydroxybenzoic acid, vanillic acid, ferulic acid, coffeic acid, syringic acid and cinnamic acid (Fig. 2 and Table 2). Owing to their functional (hydroxyl) groups, these polyphenolic compounds are known to be efficient hydrogen donors which explains a variety of biological functions [35]. In addition, **Sunny et al.** [37] verified that these functional groups found in phytoconstituents like phenolics and flavonoids is responsible for the reduction of titanium ions to TiO_2_ NPs.

**Table 2:** Polyphenolic compounds of aqueous leaf extract of *A. vera*

Compound	Concentration (μg/100 mL)
Chlorogenic acid	157.729
Rutin	58.343
*p*-coumaric acid	38.685
*p*-hydroxybenzoic acid	18.855
Vanillic acid	18.321
Ferulic acid	13.051
Caffeic acid	11.873
Syringic acid	8.362
Cinnamic acid	1.833
Protocatechuic acid	ND
Apigenin-7-glucoside	ND
Rosmarinic acid	ND
Kaempferol	ND
Chrysin	ND

*ND* Not detected

## Characterization of TiO_2_ NPs

### Morphological characterization of green synthesized TiO2 NPs

TiO_2_ NPs were prepared through an environmentally friendly technique (Fig. 1) by using *A. vera* leaves’ liquid extract, which is proved as an efficient extract for the reduction of titanium salt (TiCl_4_) to NPs [29]. The liquid extract of *A. vera* leaves was added gradually to the titanium salt solution with stirring, which results in a pinkish brown color from milky off-white after 4 h of stirring. Visual observation of color change was considered as an initial sign of synthesis, and this was in-lined with the results presented by **Satti et al.** [38].

### Spectrophotometric analysis of green synthesized TiO2 NPs

**Fig. 3:**
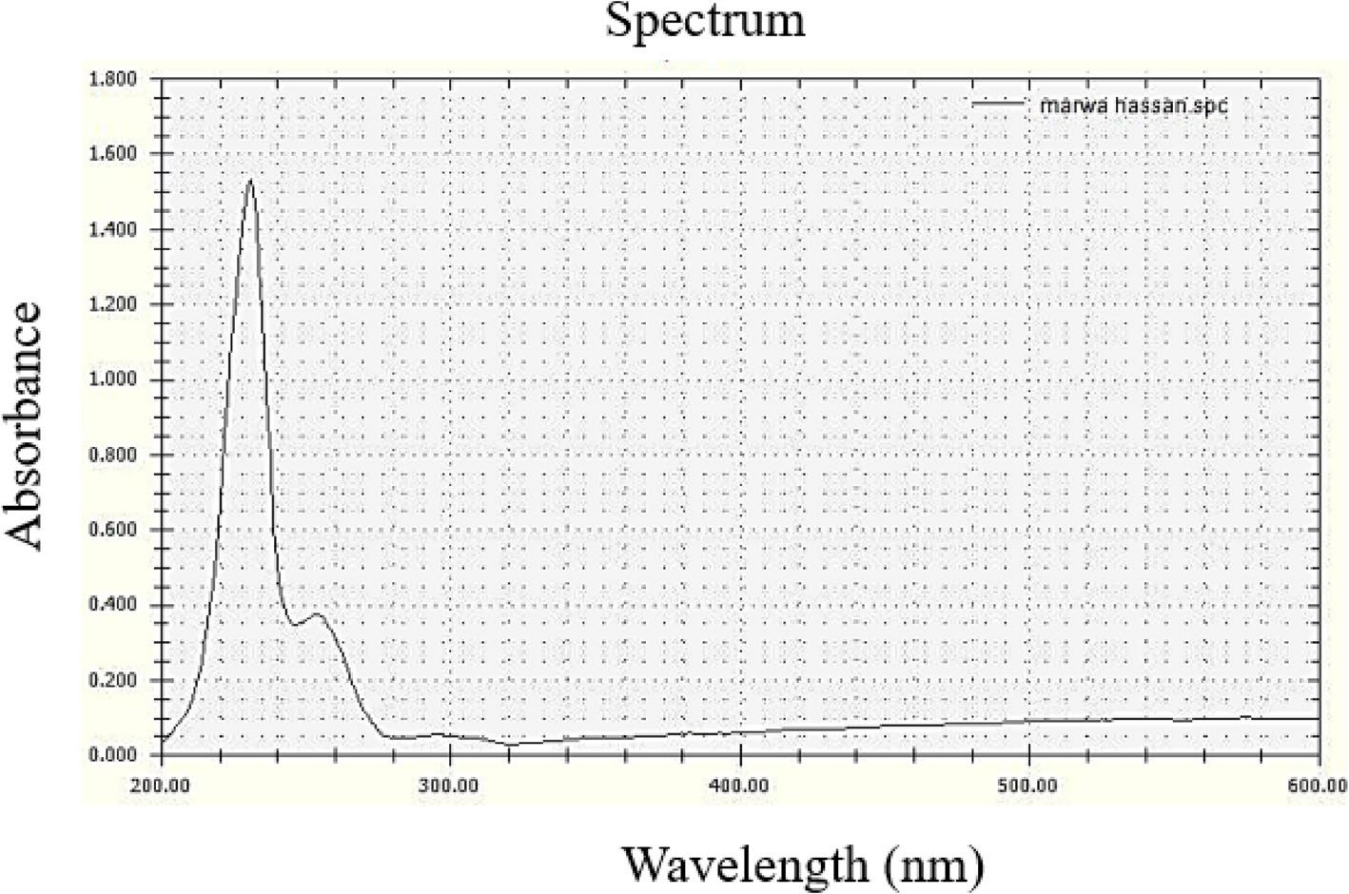
UV spectrophotometer analysis of TiO_2_ NPs

The observed change in color of titanium solution was contemplated as an affirmation of the reduction of TiCl_4_ salt into TiO_2_ NPs. The spectral analysis of the change in this color was done between the range of 200—600 nm of light wavelength. The particular peak was obtained between 200 and 300 nm (Fig. 3) which showed the formation of TiO_2_ NPs. Our results are in-lined with results of Dobrucka [39] and Satti et al. [40] who showed maximum absorption of TiO_2_ NPs at this range using *Echinacea purpurea* and *Moringa oleifera* leaf extract respectively. While those synthesized by Vijayalakshmi and Rajendran [41] and Mustafa et al. [42] revealed maximum absorption peak between 300 and 400 nm. These differences may be attributed to the sensitivity of the UV spectrum to many factors such as shape, size, and agglomeration of the particles as recently confirmed [29].

### SEM and TEM analysis of green synthesized TiO2 NPs

**Fig. 4:**
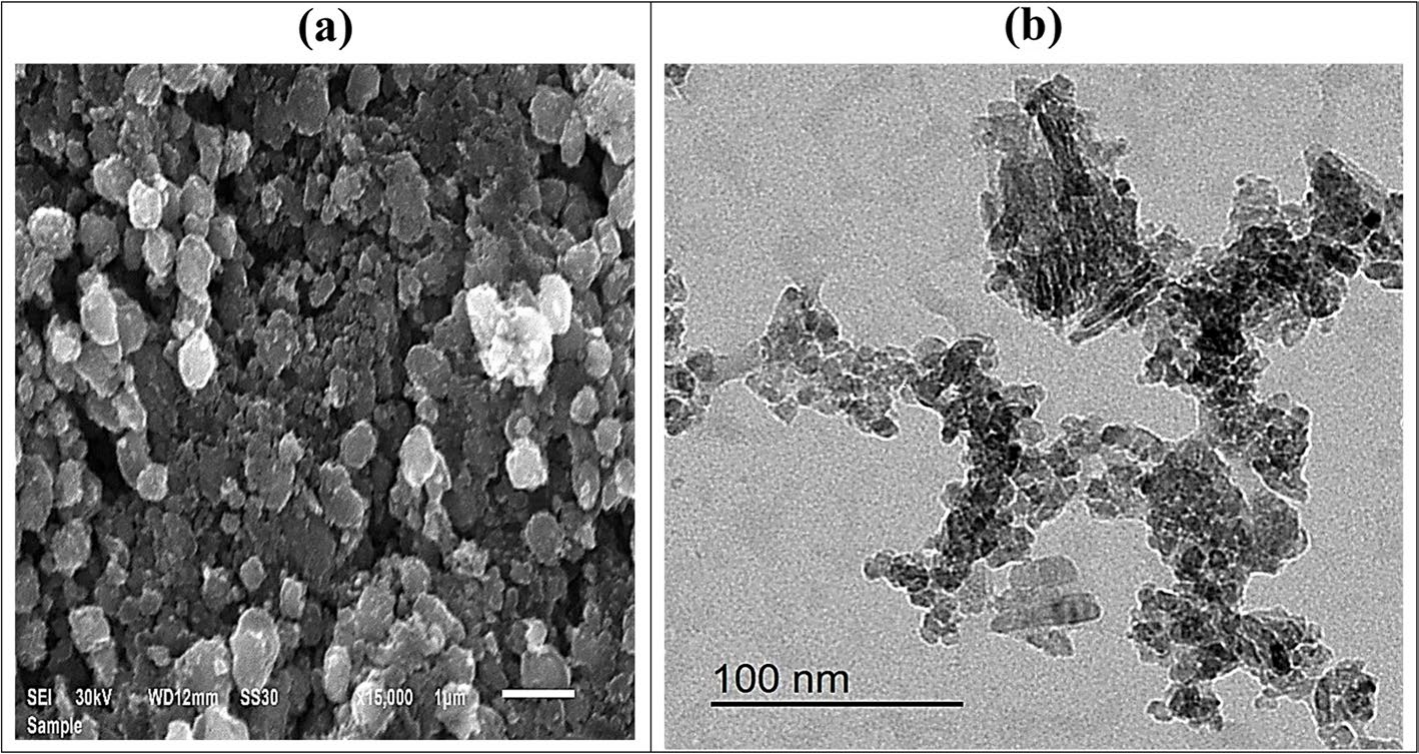
**a** SEM and **b** TEM images of green synthesized TiO_2_ NPs

Surface, size and the particle morphology of TiO_2_ NPs were imaged by SEM and TEM (Fig. 4 a, b). Based on the SEM and TEM analysis, TiO_2_ NPs showed that they are tetragonal in shape and most of the nano-forms are found in the size ranging from 10 to 25 nm (Fig. 4b). Moreover, some of the TiO_2_ NPs are fused and form tiny aggregations. Our findings are in accordance with those of Mustafa et al. [42] and Rajakumar et al. [43].

### FTIR analysis of green synthesized TiO2 NPs

**Fig. 5:**
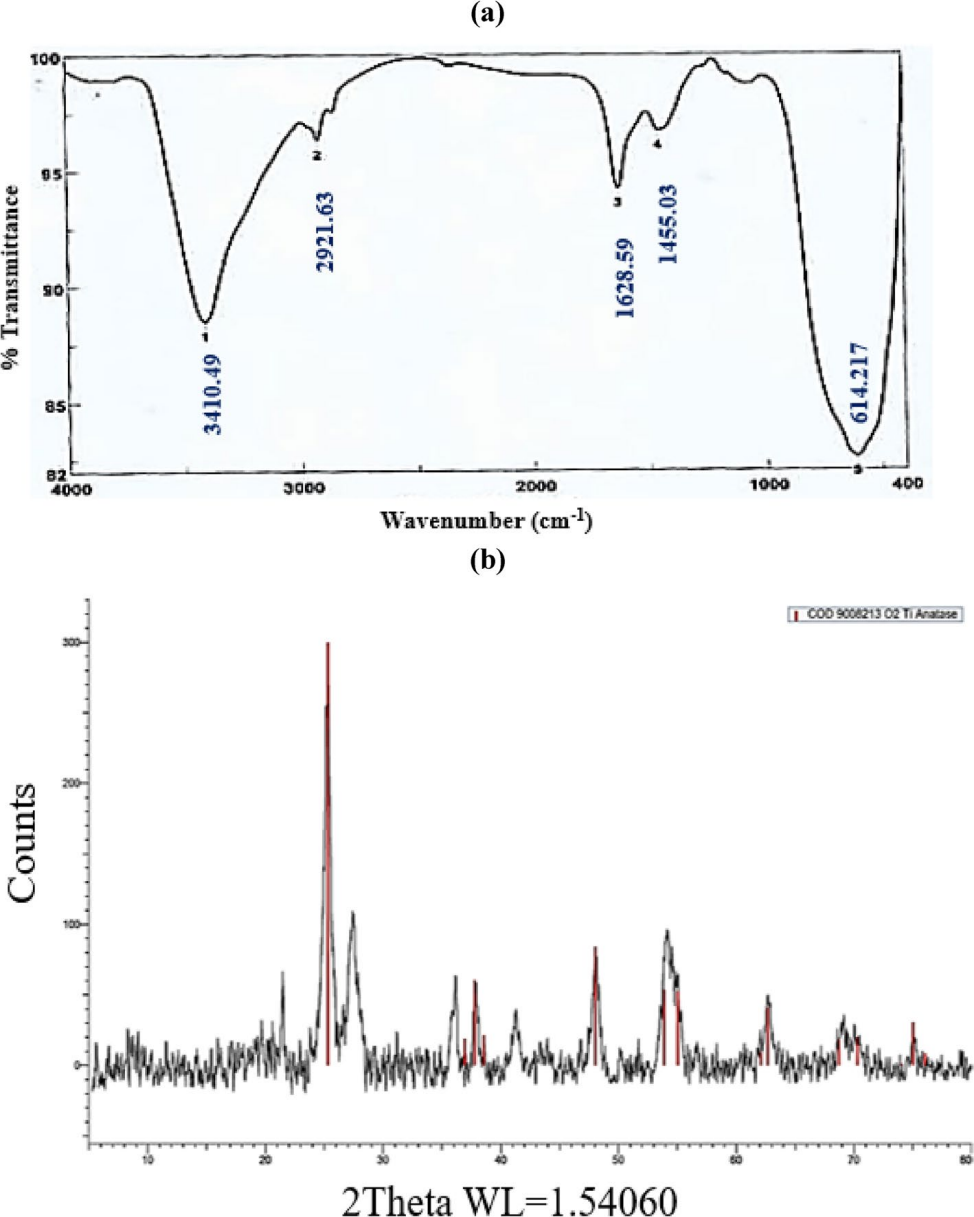
**a** FTIR spectrum and **b** XRD pattern of TiO_2_ NPs

The FTIR was performed to find out the existence of prospective phytochemical groups that were accountable for the preparation and constancy of TiO_2_ NPs. The infrared spectrum of the TiO_2_ NPs (Fig. 5a) showed a peak at 3410.49 cm^−1^ which corresponded to stretching vibrations of the hydroxyl group (-OH). The peak at 2921.63 cm^−1^ is related to C-H stretch of alkanes. The peak at 1628.59 cm^−1^ is related to -OH bending of the surface adsorbed water. The peak at 1455.03 cm^−1^ indicated the C–C stretch and C-H group. While the peak at 614.217 cm^−1^ is characteristic of Ti–O stretching and Ti–O-Ti bridging stretching modes according to Chahardoli et al. [44, 45]. These functional groups are involved in the reduction of TiCl_4_ salt into TiO_2_ NPs [40].

### XRD analysis of green synthesized TiO2 NPs

X-ray diffraction (XRD) pattern of TiO_2_ NPs was investigated to study the structure and phase formation of the sample. According to Fig. 5b, a well-crystallized anatase profile was observed for TiO_2_ NPs and the result showed that the structure was in tetragonal structure which is in a good conformity with SEM and TEM analysis. Peaks were absorbed at 25**°**, 38**°**, 48**°**, 53**°**, 62**°** and 75**°** along with miller indices values (1 0 1), (1 1 2), (2 0 0), (1 0 5), (2 0 4) and (2 1 5) respectively. The average crystallite size was measured by Debye Scherrer’s equation according to Oskam et al. [46] as 23 nm. Our results were in line with Hanafy et al**.** [29] at pH 9 (alkaline conditions) where basic TiO_2_ NPs exhibited the smallest size and composed of only one crystalline phase (anatase phase).

### Effect of TiO2 NPs on morphology and germination attributes of selected soybean varieties

**Fig. 6:**
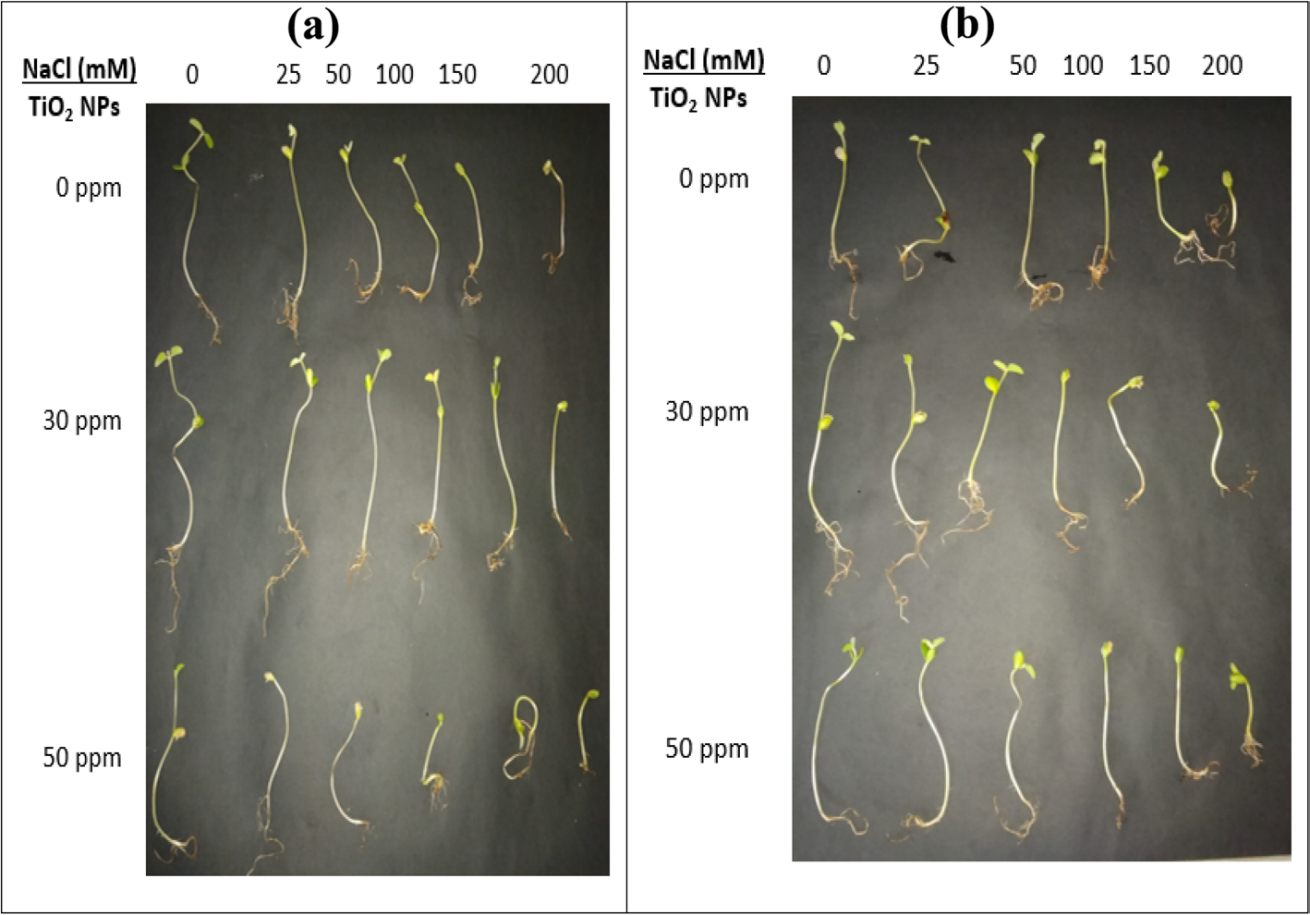
Effect of TiO_2_ NPs applications on morphological characteristic of 25-day-old soybean seedling: (**a**) var. 22 and (**b**) var. 35 under normal and salinity conditions

Critical stages for the assembly and establishment of crop plants are seed germination and early seedling growth. In the present study, different concentrations of green synthesized TiO_2_ NPs were applied to two soybean varieties (*G. max* L. var; 22 and 35) under salinity and control conditions. The effect of TiO_2_ NPs on the germination attributes of soybean varieties has been evaluated (Fig. 6a, b) and characterized as length of shoot and radicle, seedling height, radicle and shoot fwt of seedlings (Tables 3 and 4). Two-way ANOVA analysis (Table 5) revealed a significant effect of salt or TiO_2_ NPs alone or in combination (salt*TiO_2_ NPs) on all germination parameters. In both soybean varieties, seed germination and growth of seedlings were adversely affected by salinity stress, showing the lowest values at the highest NaCl concentration (200 mM). Similarly, Zafar et al. [5] confirmed that Spinach germination and development was critically reduced by salinity by reducing fresh and dry weights. The decrease in germination and growth under salt stress is due to osmotic and ion toxicity. Moreover, the uptake of nutrients and water is greatly affected under saline stress due to the reduced metabolic activity of seedlings [2, 4, 47].

**Table 3:** Effect of TiO_2_ NPs on germination parameters of soybean (var. 35) under different NaCl concentrations

TiO_2_ NPs conc	NaCl conc (mM)	Radicle length (cm)	Shoot length (cm)	Seedling height (cm)	Radicle fwt (g)	Shoot fwt (g)	Total fwt (g)
0 ppm	0	8 ± 0.212c	16 ± 0.423a	24 ± 0.635b	0.19 ± 0.005b	0.68 ± 0.017 cd	0.87 ± 0.023c
	25	7 ± 0.179d	14.5 ± 0.383bc	21.5 ± 0.569c	0.18 ± 0.0047bc	0.73 ± 0.019c	0.91 ± 0.024c
	50	5 ± 0.132f	13.7 ± 0.317 cd	18.7 ± 0.494d	0.17 ± 0.0045 cd	0.7 ± 0.018c	0.87 ± 0.023c
	100	5 ± 0.132f	12 ± 0.317e	17 ± 0.449e	0.15 ± 0.0039e	0.64 ± 0.017de	0.79 ± 0.02f
	150	3 ± 0.079i	9.5 ± 0.251f	12.5 ± 0.331f	0.13 ± 0.0034f	0.55 ± 0.015f	0.68 ± 0.017gh
	200	3 ± 0.077i	7 ± 0.185 g	10 ± 0.265 g	0.09 ± 0.0024 g	0.45 ± 0.011 g	0.54 ± 0.014j
30 ppm	0	9 ± 0.238a	17 ± 0.449a	26 ± 0.687a	0.21 ± 0.0056a	0.83 ± 0.022a	1.04 ± 0.027ab
	25	8.5 ± 0.225b	16.6 ± 0.439a	25.1 ± 0.664ab	0.22 ± 0.0058a	0.87 ± 0.023a	1.09 ± 0.029a
	50	8 ± 0.211c	15 ± 0.396b	24.50.648b	0.21 ± 0.0056a	0.78 ± 0.021b	0.99 ± 0.026b
	100	6 ± 0.159e	16.5 ± 0.316a	21 ± 0.555c	0.19 ± 0.005b	0.7 ± 0.019c	0.89 ± 0.023c
	150	4.7 ± 0.124 fg	12.5 ± 0.331e	17.2 ± 0.455e	0.17 ± 0.0045 cd	0.63 ± 0.017de	0.89 ± 0.023c
	200	3.8 ± 0.101 h	9 ± 0.238f	12.8 ± 0.331f	0.12 ± 0.0032f	0.61 ± 0.016e	0.73 ± 0.019 fg
50 ppm	0	7 ± 0.185d	14 ± 0.37bcd	21 ± 0.556c	0.13 ± 0.003f	0.72 ± 0.019c	0.85 ± 0.022e
	25	6.4 ± 0.169e	13 ± 0.344de	19.4 ± 0.513d	0.16 ± 0.004de	0.64 ± 0.017de	0.8 ± 0.021e
	50	6 ± 0.159e	12 ± 0.396e	18 ± 0.476de	0.12 ± 0.003f	0.54 ± 0.014f	0.66 ± 0.017 h
	100	4.5 ± 0.119 g	12 ± 0.317e	16.5 ± 0.436e	0.12 ± 0.0032f	0.5 ± 0.013 fg	0.62 ± 0.016 h
	150	3 ± 0.079i	9.2 ± 0.243f	12.2 ± 0.323f	0.1 ± 0.0026 g	0.45 ± 0.012 g	0.55 ± 0.0145j
	200	2 ± 0.053j	6 ± 0.159 h	8 ± 0.221 h	0.07 ± 0.0018 h	0.37 ± 0.009 h	0.44 ± 0.012 k

Data represent means ± standard errors of three biological replicates. Different letters indicate significant difference (*p* < 0·05), according to a Duncan multiple range test. fwt: fresh weight

**Table 4:** Effect of TiO_2_ NPs on germination parameters of soybean (var. 22) under different NaCl concentrations

TiO_2_ NPs conc	NaCl conc (mM)	Radicle length (cm)	Shoot length (cm)	Seedling length (cm)	Radicle fwt (g)	Shoot fwt (g)	Total fwt (g)
0 ppm	0	8 ± 0.212d	18 ± 0.476b	26 ± 0.687bc	0.22 ± 0.0058b	0.87 ± 0.023b	1.07 ± 0.0288b
	25	7 ± 0.185 fg	16 ± 0.423c	23 ± 0.608d	0.19 ± 0.005c	0.63 ± 0.0167 fg	0.82 ± 0.0216e
	50	5 ± 0.132i	14 ± 0.371d	19 ± 0.503e	0.17 ± 0.0044d	0.59 ± 0.0156gh	0.76 ± 0.021ef
	100	4.9 ± 0.129i	12.5 ± 0.331e	17.4 ± 0.461f	0.15 ± 0.0039ef	0.55 ± 0.014hi	0.7 ± 0.0185 fg
	150	3 ± 0.093 k	11 ± 0.291f	14 ± 0.371 h	0.12 ± 0.0031hi	0.5 ± 0.013i	0.62 ± 0.016 h
	200	2 ± 0.053 l	9 ± 0.238 g	11 ± 0.291i	0.09 ± 0.0024j	0.39 ± 0.0103 k	0.48 ± 0.0127i
30 ppm	0	10 ± 0.265a	22 ± 0.582a	32 ± 0.847a	0.26 ± 0.0069a	0.99 ± 0.026a	1.29 ± 0.033a
	25	9 ± 0.238b	18.5 ± 0.489b	27.5 ± 0.731b	0.2 ± 0.0053c	0.77 ± 0.021c	0.97 ± 0.0256c
	50	7.5 ± 0.198e	17.9 ± 0.474b	25.4 ± 0.672c	0.19 ± 0.005c	0.73 ± 0.019 cd	0.92 ± 0.024 cd
	100	6.6 ± 0.175 g	16.2 ± 0.429c	22.8 ± 0.603d	0.19 ± 0.005c	0.7 ± 0.0185de	0.89 ± 0.0235d
	150	5.1 ± 0.135i	14 ± 0.371d	19.1 ± 0.505e	0.17 ± 0.0045	0.64 ± 0.0169 fg	0.81 ± 0.021e
	200	3.7 ± 0.098j	12.2 ± 0.323e	15.9 ± 0.421 fg	0.13 ± 0.0034gh	0.53 ± 0.014i	0.66 ± 0.0174gh
50 ppm	0	8.5 ± 0.225c	18.4 ± 0.487b	26.9 ± 0.712bc	0.2 ± 0.0053c	0.73 ± 0.019 cd	0.93 ± 0.024 cd
	25	7.4 ± 0.196ef	16 ± 0.423c	23.4 ± 0.619d	0.16 ± 0.0042de	0.66 ± 0.017ef	0.82 ± 0.0217e
	50	5.6 ± 0.148 h	11 ± 0.291f	16.6 ± 0.439 fg	0.15 ± 0.0039ef	0.55 ± 0.0145hi	0.7 ± 0.0185 fg
	100	5.1 ± 0.135i	10 ± 0.365 fg	15.1 ± 0.399gh	0.14 ± 0.0037 fg	0.54 ± 0.014hi	0.68 ± 0.0179gh
	150	3.5 ± 0.093j	10 ± 0.265 fg	13.5 ± 0.357 h	0.11 ± 0.0029i	0.52 ± 0.0137i	0.63 ± 0.0164 h
	200	2 ± 0.053 l	9 ± 0.238 g	11 ± 0.291i	0.07 ± 0.0019 k	0.41 ± 0.0108 k	0.48 ± 0.0127i

Data represent means ± standard errors of three biological replicates. Different letters indicate significant difference (*p* < 0·05), according to a Duncan multiple range test

*fwt* Fresh weight

**Fig. 7:**
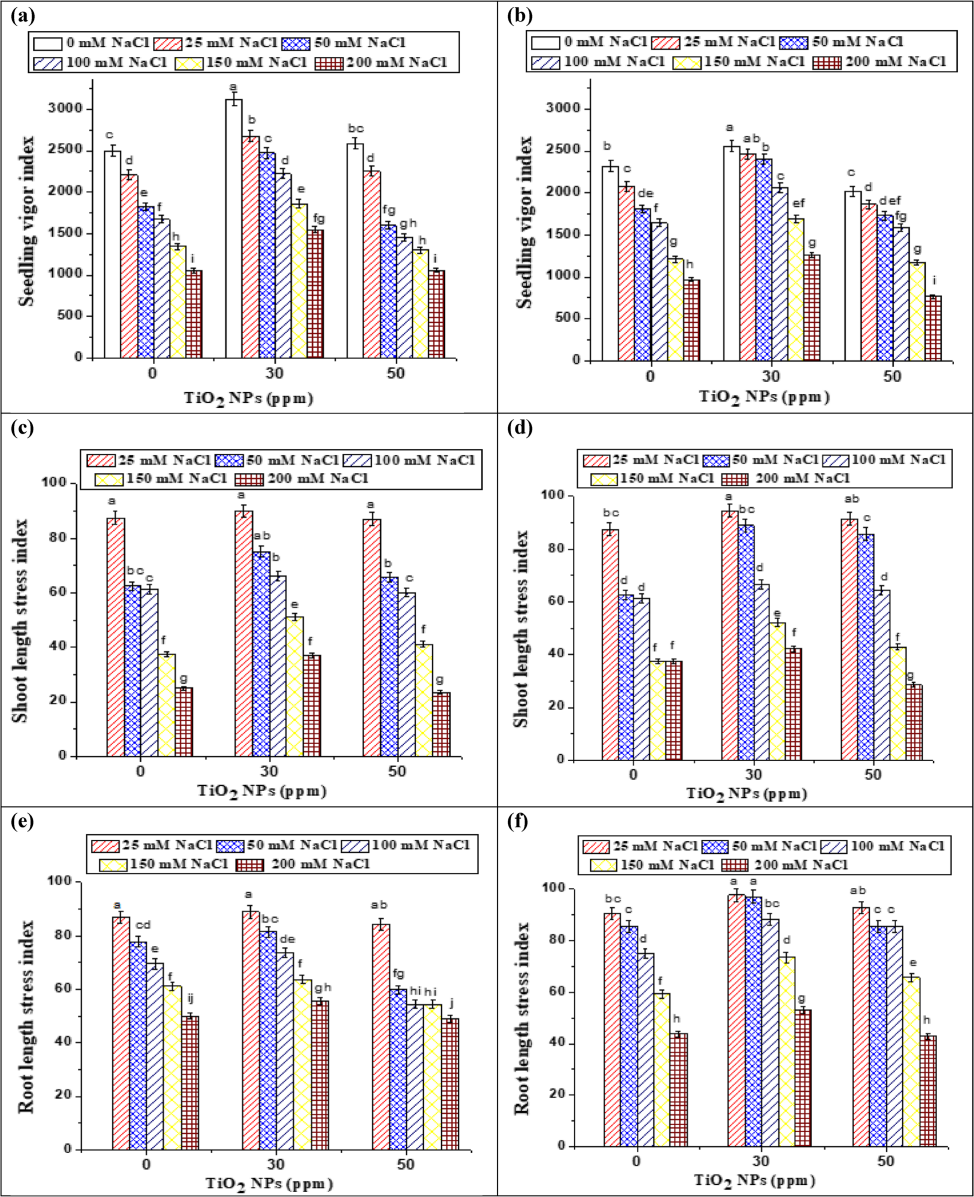
Effect of TiO_2_ NPs and different NaCl concentrations on seedling length vigor index (SLVI, **a** and **b**), shoot length stress index (SLSI, **c** and **d**) and root length stress index (RLSI, **e** and **f**) of two soybean varieties (35 and 22) respectively. Data represent means ± standard errors (error bars) of three biological replicates. Different letters indicate significant difference (*p* < 0·05), according to a Duncan multiple range test

As a crucial predictor of quick germination and seedling establishment is the seed vigor index, the present study clearly showed that seedling length vigor index (SLVI), shoot length stress index (SLSI) and root length stress index (RLSI) were significantly and dramatically reduced by saline stress at *p* > 0.05 (Fig. 7 a-f). These results showed agreement with those of Abbasi et al**.** [48] and Liu et al. [49], who described that salinity stress resulted in reduced plant vigor which could be because of the decrease in osmotic potential or ion toxicity [50].

Conversely, the priming of seeds with TiO_2_ NPs clearly enhanced the germination attributes of two soybean varieties under control and salt stressed conditions. Under control conditions, 30 ppm TiO_2_ NPs increased seedling height and total fwt by 8.3 and 19.5 percent in var 35 (Table 3), and 23.7 and 20.6 percent in var 22 (Table 4). As well, priming with TiO_2_ NPs significantly boosted the SLVI in both soybean varieties (Fig. 7a and b) which was in line with Sunny et al. [37]. In accordance with our results, several studies showed that seeds treated with TiO_2_ NPs suspensions exhibited increased germination rates, enhanced root lengths and improved seedling growth of oilseed rape [16], *G. max* [51], *Arabidopsis thaliana* [52] and cabbage [53]. Similarly, as early as 2013, a study by Raskar and Laware [54] showed that TiO_2_ NPs at concentrations ranging from 10 to 40 ppm enhanced seed germination, promptness index, and seedling growth of onions, which indicated that lower concentrations were not harmful to seed germination and early seedling growth. However, concentrations that are higher than 50 ppm could inhibit seed germination and seedling growth in onions. Most recently, Sunny et al. [37] observed that the root, shoot lengths and seedling growth of *Vigna radiate* exposed to TiO_2_ NPs exhibited a good increase compared to control. It is worth mentioning that NPs increased the seed germination and seedling growth by their ability to penetrate the seed coat, resulting in increasing water/nutrient absorption and activating the embryo [55–57]. Most specifically, Sunny et al. [37] revealed that the TiO_2_ NPs can produce more new holes during seed penetration, which are useful in transferring more nutrients efficiently, resulting in a faster germination and development rate.

Likewise, the performance was best when TiO_2_ NPs were used at 30 ppm, and a significant difference between nano-priming and salinity stress was observed as shown in Fig. 7 for SLVI, SLSI and RLSI. For instance, at 50 mM NaCl, nano-priming with 30 ppm caused a considerable increase in SLSI and RLSI (88.89 and 97.06) for soybean var 22 as compared to unprimed one (62.50 and 85.63). In accordance with our results, Shah et al. [58] showed that priming with TiO_2_ NPs has a constructive impact on the growth of maize seedlings under salinity stress conditions. This might be due to the quick completion of metabolic activities at the pre-germination stage during the priming process [59, 60], this preeminence of seed subjected to priming led to augmented seed germination levels and higher growth levels of the seedlings. According to Mahmoodzadeh et al. [16], TiO_2_ NPs improved seed germination and facilitated radicle and plumule growth in seedlings of canola crops. Jaberzadeh et al. [17] described that TiO_2_ NPs improved wheat plant development and yield-related traits under drought stress. Higher root and shoot lengths coupled with increased seedling fresh and dry weights are the attributes of early and rapid germination, resulting in higher seedling vigor.

### Oxidative damage, Reactive oxygen species (ROS) accumulation and DPPH free radical scavenging under different treatments of salinity and TiO2 NPs

**Fig. 8:**
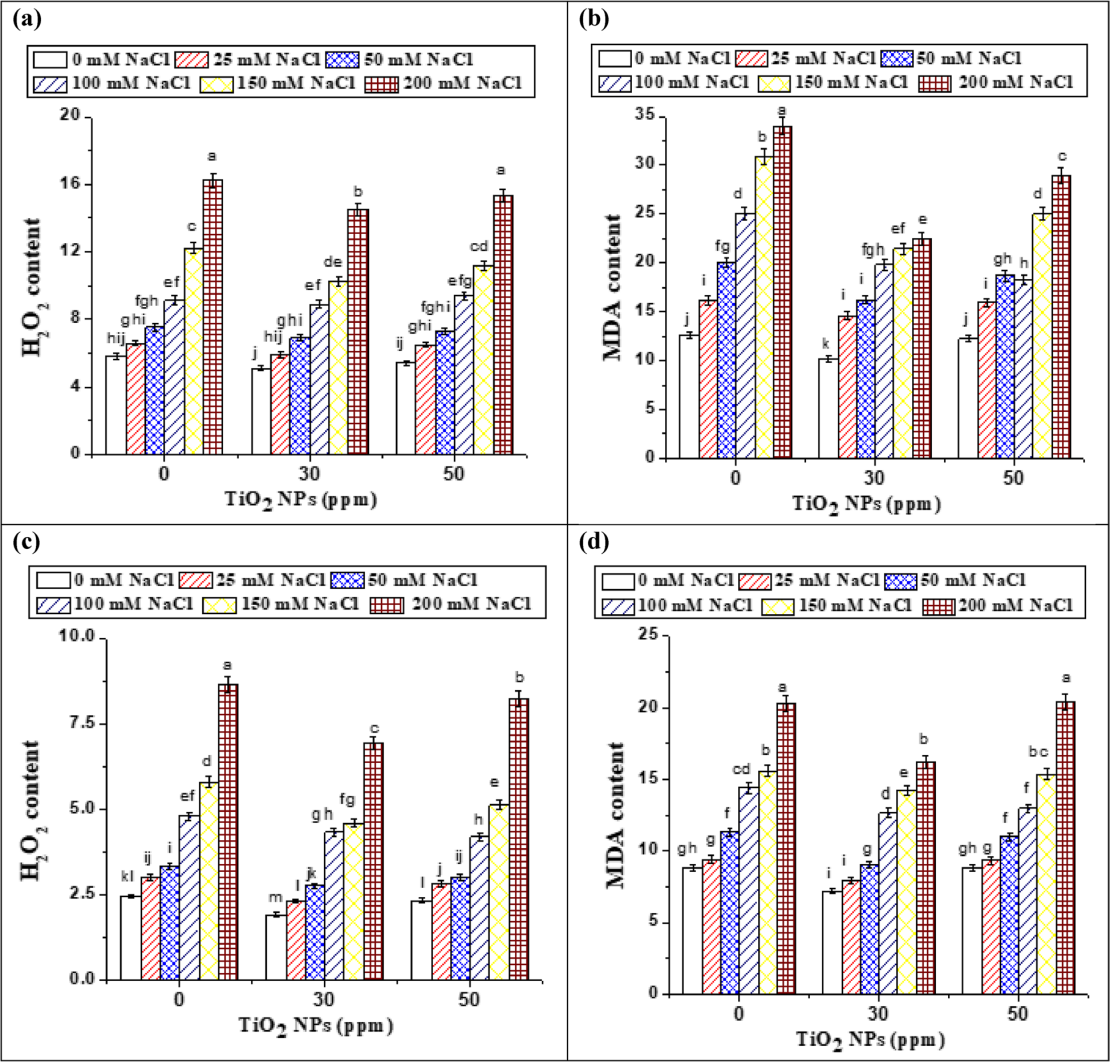
Effect of TiO_2_ NPs and different NaCl concentrations on hydrogen peroxide (H_2_O_2_, mg/g fwt) and malondialdehyde (MDA, nmol/g fwt) content of soybean var 35 (**a** and **b**) and soybean var 22 (**c** and **d**), respectively. Data represent means ± standard errors (error bars) of three biological replicates. Different letters indicate significant difference (*p* < 0·05), according to a Duncan multiple range test. fwt: fresh weight; MDA: malondialdehyde

Salinity affects plants by imposing various complications such as ion toxicity, osmotic stress, nutritional deficiency, and genotoxicity, resulting in ROS overproduction and oxidative stress [61]. The effect of salt stress on oxidative stress and lipid peroxidation was assessed in terms of H_2_O_2_ and MDA contents, which can be used as an indication to evaluate the tolerance of plants to oxidative stress as well as the sensitivity of plants to salt stress [62]. Our results showed that lipid peroxidation was influenced by salinity stress in both varieties of soybean, as confirmed by the changes in MDA content under control and treated plants as shown in Fig. 8 (b and d). In control, unprimed seedlings, an increase of 63.3 and 129.5% in MDA concentration was observed in soybean var 35 at 100 and 200 mM NaCl, respectively. The increase in MDA content under salt stress was also found in cowpea [3], fenugreek plants [4] and alfalfa [63]. In accordance with our results, data from **Jbir-Koubaa et al.** [64] suggested that salinity stress might cause a shock and photo-oxidative stress, which would cause MDA accumulation in leaves.

Moreover, our results (Fig. 8 a and c) showed a significant and gradual increase in H_2_O_2_ content by increasing NaCl concentrations. Similarly, Rehman et al. [65] discovered a 2- and threefold increase in MDA content, as well as a 2.5- and threefold increase in H_2_O_2_ production when exposed to 100 and 200 mM NaCl, respectively, illustrating salt-induced oxidative stress. Salt-stressed plants accumulate higher Na^+^ ions which cause a reduction in the water status of leaves; it may be responsible for the higher levels of H_2_O_2_ and MDA as previously mentioned by Khan [66]. H_2_O_2_ is a weak oxidizing agent and can inactivate a few enzymes directly, usually by oxidation of essential thiol (-SH) groups. Once inside the cell, H_2_O_2_ can probably react with Fe^2+^ and possibly Cu^2+^ ions to form hydroxyl radicals and this may be the origin of many of its toxic effects. It is therefore biologically advantageous for cells to control the amount of H_2_O_2_ that is allowed to accumulate [67]. Besides, Kong et al. [68] stated that MDA was an important resistant physiological index of a plant under stress.

In this respect, priming with TiO_2_ NPs reduced the level of oxidative stress and lipid peroxidation in salt-stressed plants, which was witnessed by a decrease in H_2_O_2_ content and MDA in both varieties. At the rate of 30 ppm, TiO_2_ NPs proved best and reduced H_2_O_2_ content and MDA by 11.9 and 9.6%; respectively at 25 mM NaCl stressed soybean (var 35) (Fig. 8 a and b). Most intriguingly, the beneficial effects of TiO_2_ NPs on soybean plants against salinity-induced oxidative damage may be associated with improved activity of enzymatic antioxidants, as previously reported by Abdel Latef et al. [69] on broad bean plants under salt stress and at lower TiO_2_ NP concentrations (0.01%). Moreover, Mohammadi et al. [70] and Laware and Raska**r** [71] showed that in plants treated with TiO_2_ NPs, a decrease in H_2_O_2_ level coupled with a decrease in MDA may possibly be due to the increased level of superoxide dismutase and peroxidase.

**Fig. 9:**
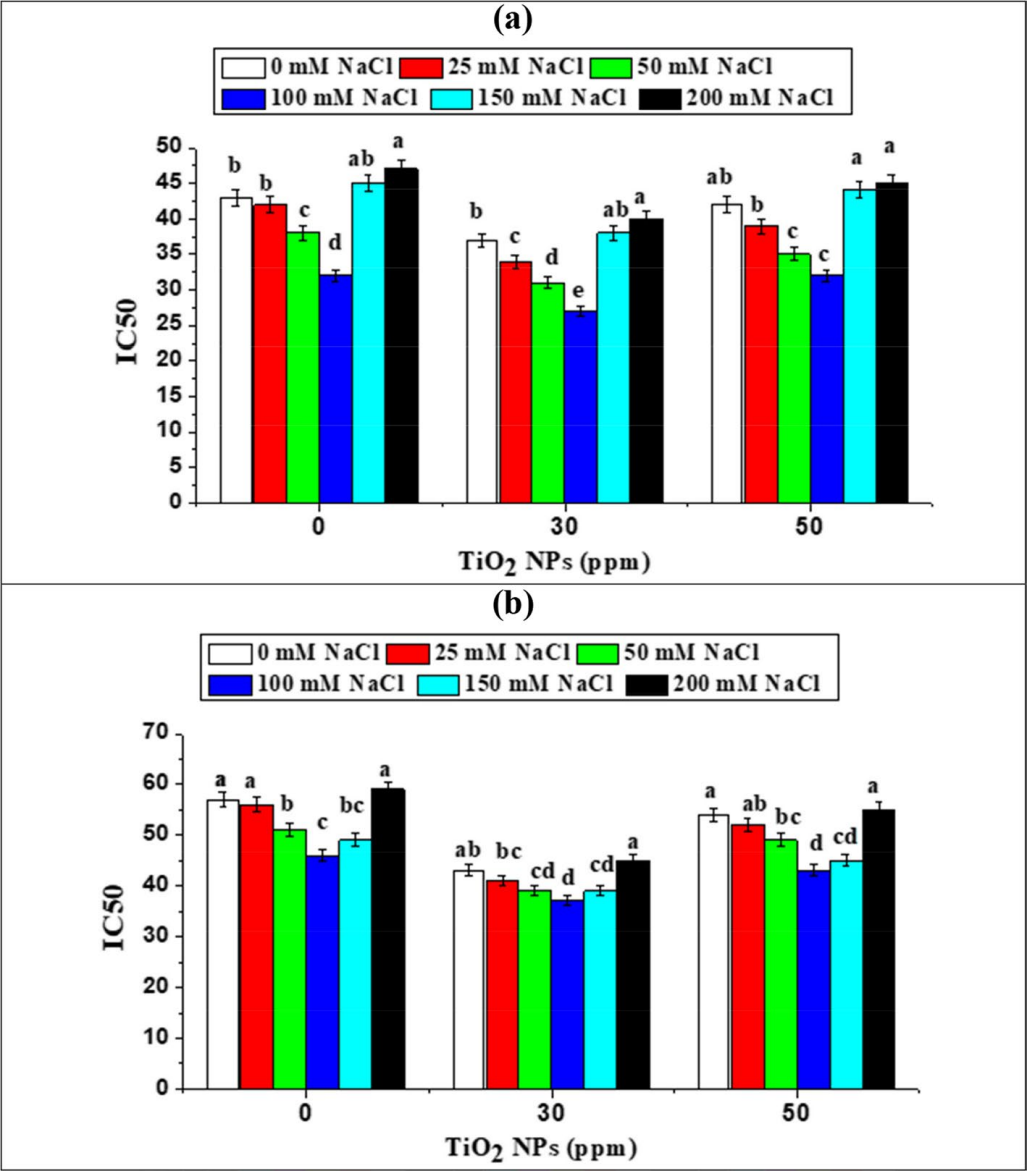
Effect of TiO_2_ NPs and different NaCl concentrations on IC50 of: (**a**) soybean var 22 and (**b**) soybean var 35. IC_50_ (the microgram of extract to scavenge 50% of the radicals)

Regulation of antioxidant machinery ameliorates the effects of salt stress in plants, as reported in many plant studies, DPPH assays are widely used to measure the antioxidant activity [72]. In this respect, in this study, antioxidant activity was determined by evaluating DPPH free radical scavenging activities and IC50 at all salt treatments. Our results (Fig. 9 a and b) showed that IC50, on basis of ANOVA results, was significantly influenced by different salt concentrations and TiO_2_ NPs applied. The antioxidant activity (DPPH free radical scavenging) of soybean seedlings increased under salt-stressed conditions. Increased antioxidant was also observed by Taârit et al. [72] in high salinity stressed (75 mM) rosette leaves. Besides, Valifard et al. [73] reported that increased level of antioxidants was found in *Salvia mirzayanii* leaves as a result of mild salinity stress. In general, plants develop protective mechanisms in response to stress by increasing the levels of antioxidants and enzymatic activities which regulate ROS levels. In this experiment, the increase in antioxidants (as stated by DPPH free radical scavenging and IC50) may be attributed to the stress induced in soybean seedlings in response to NaCl as previously mentioned by Islam et al. [74] on wheat grass. At 100 mM NaCl, leaf extracts displayed the highest DPPH free radical quenching activity (less IC50) as compared to other treatment. The results also showed that high salt-stressed (150 and 200 mM) suppresses the accumulation of antioxidants (Fig. 9). This suppression occurs under high salt-stress because high salt-stress can unbalance cellular ions, and produce active oxygen species, ion toxicity, and osmotic stress as previously mentioned by Cheeseman [75].

Most interestingly, priming with 30 ppm TiO_2_ NPs caused more enhancement in the antioxidant capacity of soybean by more DPPH free radical scavenging and lessening IC50 (Fig. 9). As a result, TiO_2_ NPs enhanced the antioxidant defense system to protect the plant against oxidative damage caused by salt stress, thus regulating ROS levels. Plants primed with TiO_2_ NPs exhibited better performance under saline conditions by increasing DPPH radical scavenging activities (decreasing IC50, Fig. 9), which correlated well with their decreased H_2_O_2_ contents and reduced levels of MDA.

### Pearson’s correlation analysis

To determine the relationship between the measured germination parameters and oxidative markers under NaCl stress and TiO_2_ NPs application, a Pearson’s correlation was performed as listed in Tables 6 and 7. There was a positive correlation between any two of germination parameters (radicle length, shoot length, seedling height, radicle fwt, shoot fwt and total fwt) for both soybean varieties. The highest correlation coefficient was that between shoot fwt and total fwt (*r* = 0.998**, *p* < 0.01) for soybean var 35 and (*r* = 0.996**, *p* < 0.01) for soybean var 22. The findings in our study were consistent with previous studies in rice and common beans [76, 77]. Moreover, these germination parameters exhibited a negative correlation with H_2_O_2_ and MDA. In contrast, H_2_O_2_ was positively correlated with MDA in soybean var 35 (*r* = 0.976**, *p* < 0.01) and soybean var 22 (*r* = 0.156*, *p* < 0.05).

**Table 5:** **Analysis** of variance (Two-way ANOVA) of the effect of salt, TiO_2_ NPs and their interactions on some germination parameters and stress markers in soybean

Variety	Soybean var. 35			Soybean var. 22		
**Parameters**	**Salt**	**NPs**	Salt*NPs	**Salt**	**NPs**	Salt*NPs
Radicle length	*	*	*	*	*	*
Shoot length	*	*	*	*	*	*
Seedling length	*	*	*	*	*	*
Radicle fwt	*	*	*	*	*	*
Shoot fwt	*	*	*	*	*	*
Total fwt	*	*	*	*	*	*
MDA	*	ns	*	*	*	*
H_2_O_2_	*	ns	*	*	*	*

*fwt* Fresh weight, *MDA* Malondialdehyde

* Significant at the *p* < 0.05; ns non-significant

**Table 6:** Pearson’s correlation matrix between seed germination parameters of soybean (var. 35) (radicle length, shoot length, seedling length, radicle fwt, shoot fwt and total fwt) and stress markers (MDA and H_2_O_2_). Each square indicates the Pearson’s correlation coefficient of a pair of parameters

**Measured parameters**	**Radicle length**	**Shoot length**	**Seedling length**	**Radicle fwt**	**Shoot fwt**	**Total fwt**	**MDA**	**H**_**2**_**O**_**2**_
Radicle length	1	0.946**	0.967**	0.861**	0.888**	0.894**	-0.884**	-0.281*
Shoot length		1	0.978**	0.934**	0.912**	0.930**	-0.852**	-0.202
Seedling length			1	0.896**	0.877**	0.893**	-0.859**	-0.238
Radicle fwt				1	0.928**	0.959**	-0.698**	-0.309*
Shoot fwt					1	0.996**	-0.741**	-0.371**
Total fwt						1	-0.741**	-0.360**
MDA							1	0.156*
H_2_O_2_								1

*fwt* Fresh weight, *MDA* Malondialdehyde

* Correlation was significant at the p < 0.05

** Correlation was significant at the p < 0.01

**Table 7:** Pearson’s correlation matrix between seed germination parameters of soybean (var. 22) (radicle length, shoot length, seedling length, radicle fwt, shoot fwt and total fwt) and stress markers (MDA and H_2_O_2_). Each square indicates the Pearson’s correlation coefficient of a pair of parameters

**Measured parameters**	**Radicle length**	**Shoot length**	**Seedling length**	**Radicle fwt**	**Shoot fwt**	**Total fwt**	**MDA**	**H**_**2**_**O**_**2**_
Radicle length	1	0.941**	0.977**	0.941**	0.930**	0.940**	-0.933**	-0.898**
Shoot length		1	0.991**	0.945**	0.952**	0.957**	-0.857**	-0.779**
Seedling length			1	0.956**	0.956**	0.964**	-0.898**	-0.836**
Radicle fwt				1	0.959**	0.976**	-0.896**	-0.853**
Shoot fwt					1	0.998**	-0.841**	-0.793**
Total fwt						1	-0.860**	-0.813**
MDA							1	0.976**
H_2_O_2_								1

*fwt* Fresh weight, *MDA* Malondialdehyde

* Correlation was significant at the p < 0.05

** Correlation was significant at the p < 0.01

## Conclusion

Ameliorating salinity tolerance and eradication of detrimental consequences of salinity are major research challenges. In the present study, salinity reduced germination and increased oxidative stress in both varieties of soybean. In this respect, the ongoing study reports the green synthesis of TiO_2_ NPs by using the reducing and stabilizing potential of *A. vera* leaf aqueous extract, which helps soybean tolerate salinity. It was discovered that 30 ppm TiO_2_ NPs is the most effective in improving germination attributes such as shoot, radicle, and whole seedling length, and fwt of shoot and radicle. Also, seed priming with TiO_2_ NPs reduced the contents of H_2_O_2_ and MDA while enhancing the DPPH free radical scavenging activities of two soybean varieties (soybean var. 35 is the most susceptible to salt stress than var. 22). It is assumed that TiO_2_ NPs have a potential to ameliorate tolerance in soybean plants against salinity. Further studies are needed to show the role of TiO_2_ NPs applications in ameliorating salinity in soybean plants under field conditions.

## Materials and methods

### *Aloe vera* plant collection and preparation of leaves extract

*A. vera* L. plant was obtained from Horticulture Department, Faculty of Agriculture, Zagazig University, Egypt after permission from Zagazig University and was identified by Dr Samir S. Teleb at the Department of Botany and Microbiology, Faculty of Science, Zagazig University according to Eggli and Newton [78]. Healthy and fresh leaves were collected and washed with tap water followed by distilled water to remove dirt and any contaminants. Following the method of Hanafy et al. [29], 250 g of the leaves were added to 1000 mL distilled water and boiled for 2 h at 90 °C. After cooling, the extract was purified by filtration using Whatman No.1 filter paper. Then the extract was filtered and stored at − 4 °C for further investigations.

### Phytochemical screening

Preliminary phytochemical screening of the *A. vera* extract was carried out to identify the active constituents, using standard methods [79, 80]**.**

### High-performance liquid chromatography (HPLC) analysis

HPLC was used to detect, identify and quantify a number of phenolic compounds in the *A. vera* extract using an Agilent Technologies 1100 series liquid chromatograph equipped with an auto sampler and a diode-array detector following a modifed method by **Kim et al. **[81]. The analytical column was an Eclipse XDB-C18 (150 X 4.6 µm; 5 µm) with a C18 guard column (Phenomenex, Torrance, CA). The mobile phase consisted of acetonitrile (solvent A) and 2% acetic acid in water (v/v) (solvent B). The flow rate was kept at 0.8 mL/min for a total run time of 70 min and the gradient programme was as follows: 100% B to 85% B in 30 min, 85% B to 50% B in 20 min, 50% B to 0% B in 5 min and 0% B to 100% B in 5 min. The injection volume was 50 µL and peaks were monitored simultaneously at 280. All samples were filtered through a 0.45 µm Acrodisc syringe filter (Gelman Laboratory, MI) before injection. Peaks were identified by congruent retention times and UV spectra and compared with those of the standards.

### Green synthesis of TiO2 NPs

The TiO_2_ NPs were prepared using TiCl_4_ as a precursor following the method of Hanafy et al. [29]. Briefly, 100 mL of *A. vera* leaves extract was added dropwise to a 100 mL of 1.0 N TiCl_4_ solution in deionized water. The mixture was kept under constant stirring for 4 h at room temperature, and the pH value was adjusted to 9 (basic) by adding NH_4_OH and HCl. The obtained suspension was filtered using Whatman No.1 filter paper to separate the formed NPs which was then washed with double distilled water repeatedly to remove the by-products and finally dried at 100 °C overnight. Further, the attained dry powder was calcined at 400 °C for 4 h to decompose all biomolecules at a high temperature where only the stable metal oxide NPs were kept [82]. Figure 1 shows a flow chart for the synthesis of TiO_2_ NPs.

## Nanoparticles characterization

The characterization of TiO_2_ NPs was done through different characterization techniques, i.e., ultraviolet (UV) visible spectrophotometer, scanning electron microscope (SEM), transmission electron microscope (TEM), Fourier transform infrared (FTIR) and X-ray diffraction (XRD).

- **The UV visible spectrophotometry** was done by using a UV–visible spectrophotometer, RIGOL (Model Ultra-3660) to verify the preparation of TiO_2_ NPs by analyzing the wavelength of the TiO_2_ NPs solution, keeping the range between 200 and 600 nm of the light wavelength.
- The morphology of the prepared particles was examined by SEM (JEOL JSM 6510 lv) and TEM (JEOL JEM-2100) at the Electron Microscope Unit (Faculty of Agriculture, Mansoura University).
- **FTIR** spectra were carried out in the wave number range of 4000 to 400 cm^−1^ using an IR spectrometer [JASCO model (FT/IR–460 Plus)] to record chemical bonds and the functional groups of the synthesized NPs.
- **XRD** was examined at room temperature on an X-ray diffractometer, using a CuKα radiation (Bruker D8 ADVANCE) in order to identify the crystal phase and to estimate the average particle size as well. It was done using a CuKα-radiation (λ = 0.154 nm). Both FTIR and XRD were carried out at the MICROANALYTICAL CENTER (Faculty of Science, Cairo University).

## Preparation of TiO_2_ NPs suspension

The suspension of TiO_2_ NPs was prepared at different concentrations of 0, 30 and 50 ppm in distilled, deionized water. The suspensions were sonicated for 4 h in a bath sonicator (Branson’s Model B200 ultrasonic) to ensure distribution of the NPs and to avoid aggregation and agglomeration.

### Seed materials, seed priming, stress treatments, and growth conditions

Different varieties of soybean were purchased after permission from Food and Legumes Research Department, Field Crops Research Institute, Agricultural Research Center, Giza, Egypt (March, 2022). Seeds of two varieties of soybean (*G. max* L. var. 22 and 35) were used in this experiment. Surface sterilization of seeds was carried out with 0.5% sodium hypochlorite for 10 min. Sterilized seeds were subjected to nanopriming of TiO_2_ NPs solution at 0 (T1), 30 (T2) and 50 (T3) ppm for 24 h at 27 °C. Following this, seeds were transferred to germination box containing sterilized sawdust, after one week of seedling emergence, NaCl with different concentrations (0 (control), 25, 50, 100, 150, 200 mM) were applied. Each treatment was replicated 3 times, for each soybean variety, a total of 54 germination boxes (18*3) were used, each containing 5 seeds. Further, all germination boxes were placed in the incubator at 98% relative humidity and 25–28 °C with dark and light photoperiod.

## Sampling

After three weeks from NaCl application, the seedlings in the germination box were washed with distilled H_2_O to remove the NaCl solution adhered to the seedlings. Then, seedlings were blotted with tissue paper to remove the water and were collected from each treatment for measuring germination parameters and the rest of the seedlings were stored for further biochemical analysis.

## Measurement techniques

### Determination of seed germination and seedling vigor indices

During the experiment, seed germination was observed, and the seed was considered as germinated after radical emergence of 5 mm. Three weeks after NaCl application, seedlings were collected for measurement of radicle and shoot length (cm) and seedling fresh weight (fwt, g). For each replication, three seedlings were randomly selected, and sample data was selected from their average values. Also, the vigor index and seedling growth stress indices [83] were calculated using the following equations:

$$\mathrm{Seedling length vigor index }\left(\mathrm{SLVI}\right)=\mathrm{Germination \% }\times \mathrm{ Seedling length }(\mathrm{cm})$$

$$\mathrm{Shoot length stress tolerance index }(\mathrm{SLSI}) \frac{\mathrm{Shoot length of treated seedling}}{\mathrm{Shoot length of control seedling}}\mathrm{x}100$$

$$\mathrm{Radicle length stress tolerance index }(\mathrm{RLSI}) \frac{\mathrm{Radicle length of treated seedling}}{\mathrm{Radicle length of control seedling}}\mathrm{x }100$$

### Quantification of ROS levels (H2O2 and MDA)

The hydrogen peroxide (H_2_O_2_) content of the seedlings was measured according to Alexievia et al. [84]. Briefly, known seedling fresh weights were homogenized with 5 mL of trichloroacetic acid (0.1% w/v) in an ice bath and then centrifuged (8000 rpm, 15 min). At that time, 0.5 mL of the supernatant was added to 0.5 mL of potassium phosphate buffer (pH 6.8, 10 mM) and 1 mL of potassium iodide (1 M). Finally, the absorbance of the mixture was recorded at 390 nm. H_2_O_2_ (mg g^−1^ fwt) content was estimated by a standard calibration curve previously made for various H_2_O_2_ concentrations.

The malondialdehyde (MDA) content was determined by homogenizing a known fresh weight of seedlings with 5 mL of 5% (w/v) trichloroacetic acid and centrifuging at 8000 rpm for 10 min [85]. Subsequently, 0.4 mL of 5% trichloroacetic acid containing 0.67% (w/v) thiobarbituric acid was added to 0.4 mL of the supernatant. The absorbance was recorded via a spectrophotometer at 532 nm and 600 nm. MDA was quantified by an extinction coefficient (155 mM^−1^ cm^−1^) and expressed as nmol g^−1^ fwt following the formula:

$$\mathrm{MDA }(\mathrm{nmol}\hspace{0.17em}\mathrm{g}-1\mathrm{ fwt}) = [(\mathrm{A}532 -\mathrm{ A}600) \times \mathrm{ V }\times 1000/\upvarepsilon ] \times \mathrm{ wt}$$

where *ɛ* is the specific extinction coefficient (= 155 mM cm^−1^), V is the volume of the extract, wt is the weight of the leaf, A is the absorbance.

### DPPH (2,2-diphenyl-1-picrylhydrazyl) radical scavenging assay

In accordance with the method of Blois [86] and Desmarchelier et al. [87], the free radical scavenging ability of the extracts was tested by the DPPH radical scavenging assay. This assay depends on the donating ability of hydrogen atoms of the sample extract which was determined by the decolorization of methanol solution of DPPH where DPPH produces a violet/ purple color in methanol solution and fades to shades of yellow color in the presence of antioxidants. A solution of 0.1 mM DPPH in methanol was prepared, and 2.4 mL of this solution was blended with 1.6 mL of extract in methanol at different concentrations. Control was prepared as above but without the sample extracts and methanol was used for the baseline correction. The reaction mixture was vortexed thoroughly and left in the dark for 30 min. Then, by using a UV spectrophotometer, the absorbance of the mixture was measured at 517 nm. Utilizing the following equation, the percentage of DPPH radical scavenging activity was computed [88]:

$$\mathrm{DPPH}\cdot\mathrm{scavengingeffect}(\%\mathrm o\mathrm f\mathrm i\mathrm n\mathrm h\mathrm i\mathrm b\mathrm i\mathrm t\mathrm i\mathrm o\mathrm n)=\{(\mathrm A0-\mathrm A1)/\mathrm A0\}\ast100$$

where, A_0_ is the absorbance of the control, and A_1_ is the absorbance of the sample extracts. % of inhibition was designed against concentration, and from the graph, IC_50_ (the microgram of extract to scavenge 50% of the radicals) was calculated. Lesser IC_50_ value shows superior antioxidant activity.

## Statistical and comparative analyses

Analysis of variance (one and two-way) was used for measuring the difference between treatments and varieties via software statistical package for the social sciences (SPSS). Significant differences in resulting data were recognized at *p* < 0.05 level by using Duncan multiple range test (95% level of probability). Analysis was carried out in triplicate and mean ± SE (standard error) of three parallel measurements. Pearson's correlation coefficients (*r*) were carried out to understand the relationship between germination traits and oxidative stress using SPSS. Figures were assembled using OriginPro 8.5 for data analysis and graphing software.

## Data Availability

All data generated or analyzed during this study are included in this published article.

## Abbreviations

DPPH: 2,2-Diphenyl-1-picrylhydrazyl
FTIR: Fourier transform infrared spectroscopy
fwt: Fresh weight
H_2_O_2_: Hydrogen peroxide
MDA: Malondialdehyde
POX: Peroxidase
*r*: Pearson's correlation coefficients
RLSI: Root length stress tolerance index
SE: Standard error
SEM: Scanning electron microscopy
SLSI: Shoot length stress tolerance index
SLVI: Seedling length vigor index
TEM: Transmission electron microscopy
TiCl_4_: Titanium tetrachloride
TiO_2_ NPs: Titanium dioxide nanoparticles
XRD: X-ray diffraction
